# Ventricular Restitution Predicts Paroxysmal Atrial Fibrillation in Horses

**DOI:** 10.1093/function/zqaa038

**Published:** 2020-12-09

**Authors:** Julia Ramírez, Andrew Tinker

**Affiliations:** Clinical Pharmacology, William Harvey Research Institute, Barts and The London School of Medicine and Dentistry, Queen Mary University of London, London, EC1M 6BQ, UK

## A Perspective on “ECG restitution analysis and machine learning to detect paroxysmal atrial fibrillation: insight from the equine athlete as a model for human athletes”

In this issue of *FUNCTION*, Huang et al. extract RR, QT, and TQ intervals measured from equine ECG recordings with and without paroxysmal atrial fibrillation (PAF).^1^ The authors then use a machine learning algorithm (k-nearest neighbors [kNN]) to discriminate and classify PAF cases. They show that it is possible to do so indicating a link between ventricular electrical function and atrial arrhythmia. Furthermore, performance was enhanced by including all three parameters (RR, QT, and TQ) in the analysis.

Atrial fibrillation (AF) is characterized by an irregular electrical activity of the atria and is the commonest arrhythmia in man resulting in significant morbidity and mortality. However, AF also occurs in other species and is the most prevalent arrhythmia affecting performance in thoroughbred horses. Although AF can be detected by performing an electrocardiogram (ECG), there are problems when the fibrillatory events are paroxysmal occurring before or after periods of normal sinus rhythm so-called PAF. Horses are particularly predisposed to PAF and conversion to sinus rhythm usually leads to a return to their prior performance level.^2^ Several methods have been proposed in the medical literature using ECG analysis to indirectly detect the potential for PAF.^3^ Huang et al.^1^ addressed this question and examined if ECG markers of ventricular electrical restitution measured from the surface ECG could potentially be associated with PAF using horses as a model.

**Figure 1.**
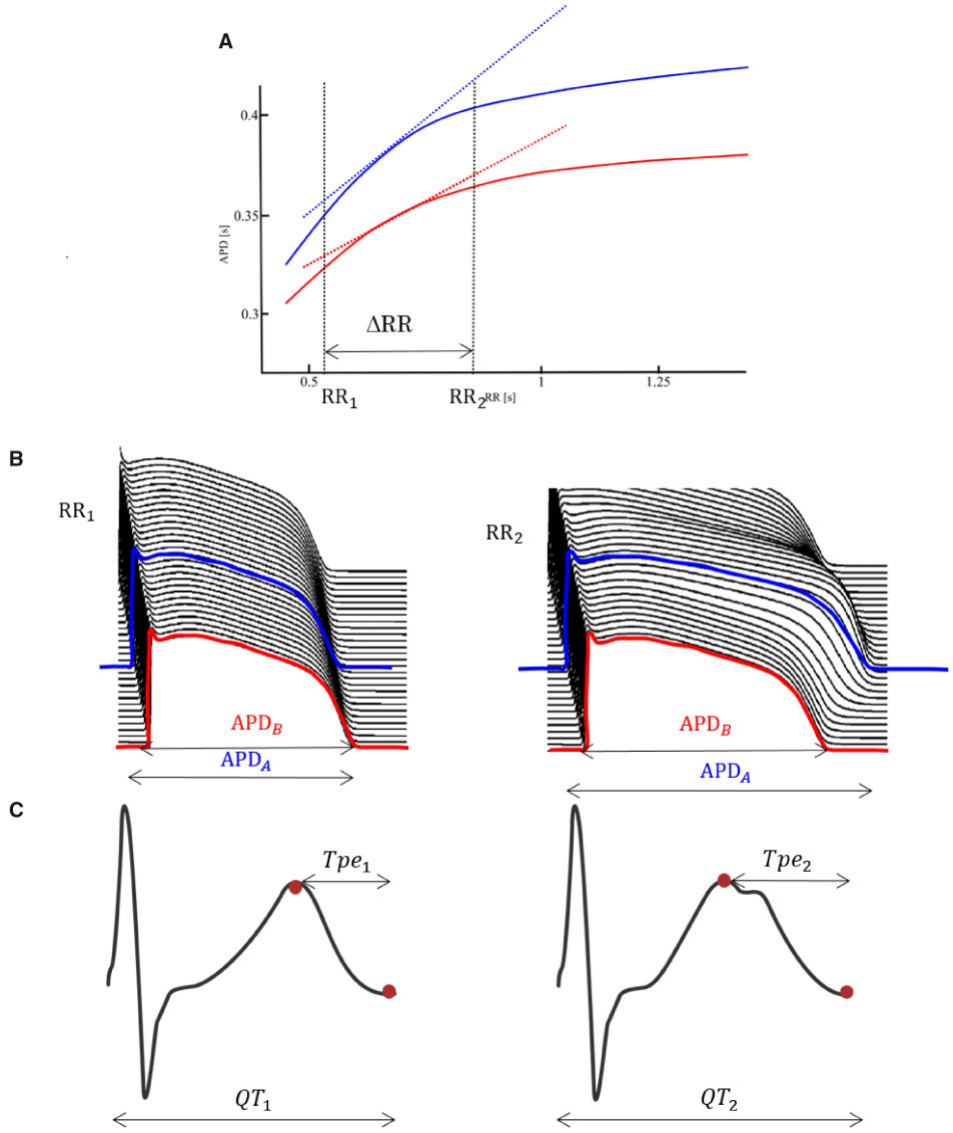
Electrical Restitution. (**A**) Two APD restitution curves, depicting the APD as a response to the preceding RR interval, of two cardiomyocytes, A (blue) and B (red, **B**). The slope of the APD restitution curve measures the change in APD with respect to a change in RR, and high values have been associated with increased arrhythmogenic predisposition. These changes in the APD are reflected on the ECG signal, as shown in (**C**).

Electrical restitution refers to mechanisms whereby the electrical activity of the heart responds to a variation in heart rate (HR) and changes in electrical restitution may be associated with arrhythmic risk. The action potential duration (APD) restitution curve represents the APD of a cardiomyocyte as a function of the preceding RR interval (inverse of HR) and is usually almost flat for RRs longer than 1 s in man and steeper for shorter RRs.^4^Figure 1A shows the APD restitution curves of the two myocytes (A and B) depicted in Figure 1B. The slope of the curves between two given RR interval values, RR1 and RR2, reflect the change in each respective APD as a response to the change in RR. Electrical dysfunction can cause the APD of a cardiomyocyte (such as A in Figure 1B) to abnormally prolong as a response to the change in RR, leading to a steep and potentially proarrhythmic APD restitution curve. The repolarization time of a cardiomyocyte is influenced by the time required for the impulse to propagate to and depolarize that myocyte, i.e., conduction velocity, and the cardiomyocyte’s intrinsic APD. Repolarization time differences, or dispersion of repolarization, normally exist between cells of different chambers of the heart and between myocytes of different regions within the ventricular wall and is influenced by the dispersion of APD, i.e., the difference between the APDs of different cardiomyocytes.^5^ Dispersion of repolarization also has a dependence on the RR interval, with the dispersion decreasing as RR decreases. This concept is illustrated in Figure 1A. Due to restitution kinetics of dispersion of repolarization, the two curves exhibit differences in range and slope across the RR interval values. Several studies have shown that increased dispersion of repolarization restitution also facilitates arrhythmia development.^6^ Restitution can be studied using surrogates of APD and repolarization such as the activation recovery interval measured from cardiac electrograms or ECG parameters such as the QT and TQ interval (Figure 1C), which equate to repolarization time and diastolic interval, respectively.

It has been demonstrated that hearts with AF typically have larger atrial volumes, as well as a much larger variation in volume compared to healthy hearts. Once present, AF results in the loss of synchronized atrial contraction, which affects ventricular filling, atrial reservoir, and conduit function, and the function of atrioventricular valves. Importantly, ventricular remodeling also occurs as a consequence of AF, and studies have demonstrated this remodeling can be reversible after successful restoration of sinus rhythm.^7^ AF-induced remodeling may alter the restitution properties mentioned above and therefore be reflected in the ECG. Indeed, Huang et al.^1^ hypothesized that these restitution changes are an indicator of electrical alterations that exist following the development of PAF. A recently proposed ECG marker of electrical restitution is T-wave morphology restitution (TMR),^8^ which is strongly associated with ventricular arrhythmias. However, TMR was also demonstrated to be significantly associated with AF in asymptomatic individuals in sinus rhythm,^8^ supporting Huang et al.’s hypothesis.

The simplest way to measure the above restitution markers is to divide the intervals reflecting the electrical event of interest, i.e., QT, by the preceding RR interval. Huang et al.,^1^ instead, used machine learning to automatically extract patterns from the relationship between TQ, reflecting atrial activity, QT, reflecting ventricular activity, and RR, reflecting autonomic activity, that could be associated with PAF. The algorithm the authors used is kNN, which is one of the simpler machine learning models. Alternative algorithms have an increased classifying power so future studies could explore the selection of the optimal machine learning algorithm. More advanced techniques, like neural networks, have widened the spectrum of ECG analysis, automatically identifying the most important aspects or features of the ECG signal contributing to AF.^9^ However, as the complexity of the algorithm increases, outputs algorithms become more difficult to interpret and hence remain treated as “black-boxes,” where no information regarding the underlying mechanisms or risk is explicitly generated. A second important extension of the work would be to increase the number of PAF cases which would help develop the algorithm and avoid the random resampling approaches used in the paper for the training and test datasets.

It is known that elite endurance human athletes have an increased predisposition to AF.^10^ Huang et al. in this issue^1^ have assessed the use of an equine equivalent as a model for human athletes, as there are currently no open access databases of such in man. The latter is likely to change with the increased use of telemedicine and development of smart devices monitoring HR. Their work is an important contribution and future studies will confirm the validity of their findings in human athletes.
